# Age‐Dependent Increase in Small Intestinal Permeability and Sex‐Dependent Absorptive Capacity in Cats (*Felis catus*)

**DOI:** 10.1111/jpn.70015

**Published:** 2025-11-02

**Authors:** Keely Patterson, Emma N. Bermingham, Karl Fraser, Daniel Bernstein, Karin Weidgraaf, Anna K. Shoveller, David G. Thomas

**Affiliations:** ^1^ School of Agriculture and Environment Massey University Palmerston North New Zealand; ^2^ AgResearch Ltd Private Bag Palmerston North New Zealand; ^3^ Fonterra Dairy Farm Road, Fitzherbert Palmerston North New Zealand; ^4^ Riddet Institute Palmerston North New Zealand; ^5^ Centre for Feline Nutrition Massey University Palmerston North New Zealand; ^6^ Department of Animal Biosciences University of Guelph Guelph N1G 2W1 Canada

**Keywords:** digestibility, gut barrier function, gut permeability, LC‐MS, leaky gut, mucosal barrier, sugar probe

## Abstract

Age‐associated changes in intestinal permeability and function have not been studied in domestic cats, leaving a key factor in the relationship between age and digestive health in cats unexplored. Due to factors not currently understood, mature and senior cats may experience a loss of fat and protein digestibility, along with a loss of body weight (BW), impacting lifespan and quality of life. Therefore, to establish the relationship between age and intestinal health, intestinal permeability and absorptive capacity were quantified in young and senior cats using a differential sugar absorption test (SAT) on cat plasma. A solution containing four different sized sugars was orally administered to 36 healthy mixed‐breed domestic shorthair cats (male (*n* = 21) and female (*n* = 15)) split into two groups by age, young 2.40 ± 0.758 (*n* = 21) and senior 11.23 ± 1.896 (n = 15) years (mean ± SD). Blood was collected before and again 3 h after dosage and plasma was analysed using liquid chromatography mass‐spectrometry (LC‐MS). Intestinal permeability was higher (*p* = 0.004) in senior cats than young cats, and was not affected by sex (*p* = 0.288), sampling date (*p* = 0.652), or BW (*p* = 0.951). Absorptive capacity was higher (*p* = 0.033) in male cats than females, and was not affected by age class (*p* = 0.440), sampling date (*p* = 0.580), or BW (*p* = 0.652). In conclusion, intestinal permeability was higher in older cats and suggests age‐related changes in intestinal barrier structure and function. These findings highlight the need to further consider increased intestinal permeability as a cause of reduced nutrient digestibility in older cats, offering a new target for interventions to enhance their health and well‐being.

## 1 Introduction

The integrity and functionality of the intestinal barrier can be characterised by its permeability and absorptive capacity. A healthy gastrointestinal tract (GIT) epithelium maintains low permeability to maintain selective absorption of molecules, while limiting the entry of potentially harmful substances, such as pathogens and bacteria (Craven et al. 2007). Permeability of the intestinal barrier is managed by tight junctions (TJ), specialised intercellular structures between epithelial cells that control paracellular transport and help preserve the barrier's integrity and function (Bischoff et al. 2014). Tight junction proteins control the paracellular pathway, the transport of hydrophilic molecules and water between epithelial cells, while cell surface transporters control the transcellular pathway, the transport of hydrophilic or lipophilic molecules through the cells (Farré et al. 2020; Vanuytsel et al. 2021). Another characteristic of the intestinal barrier is its mucosal absorptive capacity. This measurement assesses the efficacy of carrier‐mediated active transport pathways, primarily used to absorb dietary sugars and other substances from the digestive process (Rajan et al. 1961; Johnston et al. 2001).

The impacts of age on intestinal permeability have not been studied in cats. In rats, mice, and baboons, studies indicate an increase of intestinal permeability with age (Katz et al. 1987; Ma et al. 1992; Tran and Greenwood‐Van Meerveld 2013; Thevaranjan et al. 2017). On the other hand, contrasting findings in humans claim the absence of age‐related effects (Wilms et al. 2020). Increased intestinal permeability, and thus a weakened mucosal barrier, has been linked to disease, and has been shown to promote inflammation, influence the gut microbiome, and overall weaken the protective effects of the mucosal barrier (Bischoff et al. 2014). Cats have increasing risk of decreased fat (Anantharaman‐Barr et al. 1991; Harper 1998; Peachey et al. 1999; Perez‐Camargo 2003; Fahey et al. 2008; Patil and Cupp 2010; Teshima et al. 2010; Salas et al. 2014) and protein (Harper 1998; Perez‐Camargo 2003; Patil and Cupp 2010; Teshima et al. 2010) digestibility with age. Evaluating the relationship between age and intestinal permeability will improve basic knowledge of the GIT of the domestic cat and allow researchers to study the possible cause of reduced GIT function in senior cats.

One method of quantifying intestinal permeability in vivo is through the differential sugar absorption test (SAT). This method employs disaccharides and monosaccharides or polyalcohols to measure paracellular and transcellular permeability, respectively, of the small intestinal mucosal barrier (Lostia et al. 2008). Normally, the TJs that line the intestinal barrier do not allow a high concentration of disaccharides as opposed to monosaccharides to permeate through, creating a low ratio of disaccharides to monosaccharides, commonly lactulose to mannitol (LM) or lactulose to rhamnose (LR) (Bischoff et al. 2014). When the intestinal barrier is compromised, the concentration of disaccharides able to pass through the barrier increases, therefore increasing the numerical value of the ratio. Absorptive capacity of the intestine can also be determined using a SAT with different sugars: Xylose and 3‐O‐methylglucose (3‐OMG) can be used to assess carrier‐mediated active transport and ATP‐dependent mediated active transport, respectively (Rodríguez et al. 2009). A healthy intestine will maintain a higher xylose to 3‐OMG (XG) ratio, signifying their ability to actively transport molecules across the membrane (Craven et al. 2007). A pilot study was conducted to establish a minimally invasive method to measure small intestinal permeability and absorptive capacity in the domestic cat, whereby the sugars (‘sugar probes’) lactulose, rhamnose, xylose, and 3‐OMG were administered to 13 male young adult cats. The dose described in this study was sufficient to allow detection of the sugar probes in both plasma and serum at 180 min (Patterson et al. 2024).

Advancing age is hypothesised to increase intestinal permeability (increasing LR ratio) and decrease absorptive capacity (decreasing XG ratio). Therefore, the objectives of the present study were to determine if there are any differences in intestinal permeability and absorptive capacity between young and late midlife to super‐senior cats, defined by Salt et al. (2023), which are classified as youth and senior groups in this paper.

## 2 Materials and Methods

### 2.1 Ethics

This study was approved by the Massey University Animal Ethics Committee (MUAEC 23/14), New Zealand which meets the requirements of the Animal Welfare Act (1999).

### 2.2 Animals, Diets, and Housing

Thirty‐seven healthy male and female domestic shorthair cats from the Centre for Feline Nutrition at Massey University, Palmerston North, New Zealand were selected based on age and sex (Table 1). Due to the nature of the research colony, all male cats are neutered at approximately 6 months of age and the majority of female cats are entire. One young, female cat did not give enough blood for a complete blood count (CBC), so her health was determined based on previous blood samples and a physical check. Data from one senior male cat was omitted due to problems blood sampling and a resulting 216‐min gap in between T0 and T3 blood samples. Cats in this colony are monitored on a daily basis for behaviour and stool quality, weighed on a weekly basis, and are up to date with appropriate vaccinations. A CBC was taken to ensure the older cats were healthy and these were used as explanatory values in the analysis.

**Table 1:** Cat data.

	Male senior	Male youth	Female senior	Female youth
*n*	8	13	7	8
Age (years) Mean ± SD (range)	11.53 ± 2.316 (9.23–16.21)	2.48 ± 0.845 (1.26–3.37)	10.88 ± 1.364 (9.48–12.53)	2.26 ± 0.616 (1.26–3.37)
BW (g) Mean ± SD (range)	3934.82 ± 383.016 (3536.10–4415.30)	4238.79 ± 514.716 (3358.60–5079.90)	3112.36 ± 334.414 (2796.60–3745.80)	3193.89 ± 370.784 (2609.60–3540.50)
N:E	8:0	13:0	3:4	0:8

Abbreviations: BW, body weight; N:E, neutered:entire.

**Figure 1:**
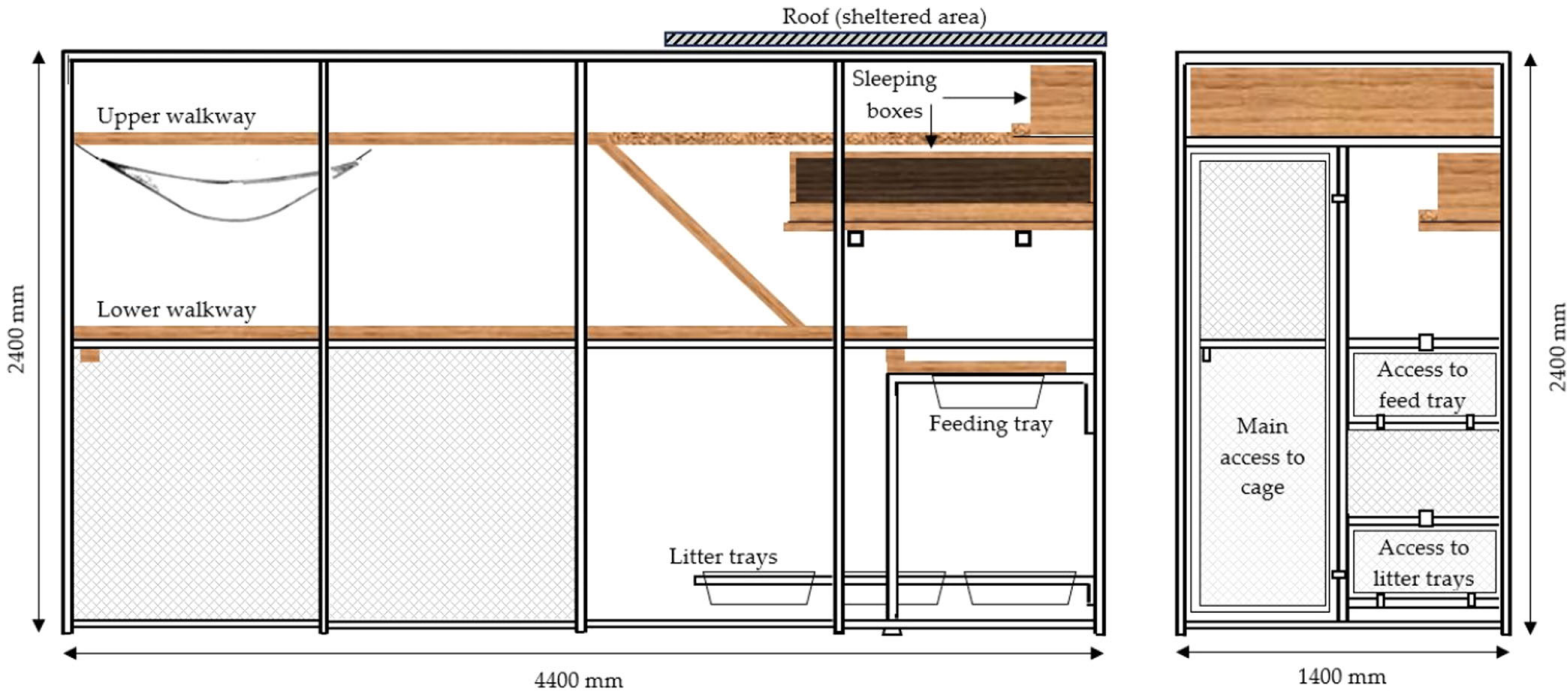
Colony cages at Massey University's Centre for Feline Nutrition, in mm. [Color figure can be viewed at wileyonlinelibrary.com]

As part of normal husbandry, cats were fed ad libitum in their group enclosure (Figure 1 developed by (Smit et al. 2023)) with multiple flavours of a commercially available retorted diet (Heinz Wattie's Ltd. Hastings, New Zealand) formulated to meet the Association of American Feed Control Officials (AAFCO) requirements for adult cats in colony cages and always had ad libitum access to fresh water.

### 2.3 Sample Collection

The cats were placed in individual metabolic cages (measuring 80 × 80 × 110 cm; as previously described in Hendriks et al. 1999) for a 12 h fasting period before the initial blood sample was collected with only access to water. This fasting period was implemented to confirm the cats' physical well‐being through a CBC and to establish a sugar‐free baseline. At the end of the 12 h fast, cats were weighed, 2 mL of blood via jugular venepuncture was taken for a baseline sample (T0) for sugar probe analysis and a CBC, and then cats were dosed with a sugar probe mix at 2 mL/kg BW (body weight). The probe mix was formulated to contain 0.07 g/mL lactulose, 0.02 g/mL rhamnose, 0.07 g/mL xylose, and 0.02 g/mL 3‐OMG dissolved in purified water (Patterson et al. 2024).

Cats were returned to their enclosure for 3 h with no access to food and ad libitum access to water. After 3 h, blood sampling was repeated on the opposite jugular vein, again taking 2 mL of blood (T3). Once the final blood sample was taken, the cats were returned to their colony cages with access to their normal diet. One mL of the T0 blood was transferred to a 3.6 mL K3‐EDTA tube (Becton, Dickinson and Company, Franklin Lakes, NJ, USA) for CBC. The rest of the T0 blood, and all the T3 whole blood was transferred to a 5.4 mg K2‐EDTA tube (Becton, Dickinson and Company, Franklin Lakes, NJ, USA) and spun at 3000 × g at 4°C for 15 min. Plasma was then aliquoted into microcentrifuge tubes for LC‐MS and frozen at −80°C until analysis. Due to the number of animals, the sugar probe testing and blood sampling was conducted over three consecutive days.

### 2.4 Sample Preparation and Analysis

Haematology (CBC) values were analysed by IDEXX using a ProCyte Dx Haematology Analyser (IDEXX Laboratories Pty. Ltd. Tennant Drive, PO Box 325, Palmerston North 4440, New Zealand) the same day blood was collected. Plasma was analysed for reticulocyte haemoglobin equivalent (retic Hb), erythrocyte count (RBC), haemoglobin (Hb), haematocrit (HCT), mean cell volume (MCV), mean corpuscular haemoglobin (MCH), mean corpuscular haemoglobin concentration (MCHC), platelet count, absolute reticulocyte count, blood leukocyte count (WBC), band neutrophil count, segmented neutrophil count, lymphocyte count, monocyte count, eosinophil count.

The sugar probes sample preparation and quantification was performed as previously published (Patterson et al. 2024). Briefly, plasma samples (200 µL) were mixed with ice cold acetonitrile (ACN; 590 µL) and internal standard mixture (10 µL containing all four 13C‐labelled sugars), vortexed thoroughly, incubated (−20°C, 60 min), vortexed again, and centrifuged (14,000 × g, 10 min, 4°C). Then, 100 µL of Milli‐Q® water was added to each sample, vortexed, and centrifuged (14,000 × g, 10 min, 4°C) for a second time. A fixed volume of the supernatant (800 µL) was dried under nitrogen at 35°C, and the dry extract was reconstituted in 100 µL of 90% ACN with 1 mM ammonium formate.

### 2.5 Liquid Chromatography‐Mass Spectrometry

The LC‐MS method used to measure the concentration of the sugars in the plasma has been previously described (Patterson et al. 2024). Analyses were conducted over two consecutive days and for both days, a pooled sample of a homogenous mixture of an aliquot of each cat plasma sample was analysed as a pooled quality control (PCQ) and repeats of the mid‐range calibration standard were analysed as a technical quality control (TQC). Limit of detection (LOD) and limit of quantitation (LOQ) were assessed using the same protocol as described by Patterson et al. (2024). Linearity and quality control measures are summarised in Table 2.

**Table 2:** Data collected from calibration curves.

Analyte	Equation	R^2^	PQC_RSD (%)_	TQC_RSD (%)_
Lactulose	y = 0.187873*x* – 0.11872	0.9989	Day 1—2.914	1.769
			Day 2—3.521	
Rhamnose	y = 0.130345*x* – 0.0315225	0.9997	Day 1—4.084	2.144
			Day 2—5.956	
Xylose	y = 0.0660358*x* – 0.00774623	0.9999	Day 1—0.855	1.334
			Day 2—1.147	
3‐OMG	y = 0.159498*x* – 0.0839806	0.9997	Day 1—2.771	0.998
			Day 2—2.345	

Abbreviations: PQC, pooled sample quality control; RSD, relative standard deviation; TQC, technical quality control; 3‐OMG, 3‐O‐methylglucose.

### 2.6 Equations

Sugar concentrations were calculated based on equations provided in Patterson et al. (2024), then divided by the amount of sugar ingested to normalise for the dosage. Equations used to calculate LR and XG ratios are shown in Equations (1) and (2), respectively, using the concentration of the sugar quantified in the plasma and the amount of the sugar ingested by each cat in the sugar solution.

(1) $$L:R=\frac{\left(\mathrm{Lactulose Recovered}\right)}{\left(\mathrm{Rhamnose Recovered}\right)}=\frac{\left(\frac{\mathrm{Lactulose Concentration}}{\mathrm{Lactulose Ingested}}\right)}{\left(\frac{\mathrm{Rhamnose Concentration}}{\mathrm{Rhamnose Ingested}}\right)}$$

(2) $$X:G=\frac{\left(\mathrm{Xylose Recovered}\right)}{\left(3-\mathrm{OMG Recovered}\right)}=\frac{\left(\frac{\mathrm{Xylose Concentration}}{\mathrm{Xylose Ingested}}\right)}{\left(\frac{3-\mathrm{OMG Concentration}}{3-\mathrm{OMG Ingested}}\right)}$$

### 2.7 Statistical Analysis

Certain CBC data were omitted from the analysis as they were identified as outliers according to Chauvenet's criterion.

All statistical analyses were carried out using RStudio version 4.1.1. Correlations between haematology data and age as well as haematology data and intestinal permeability data were assessed using the Pearson correlation. Intestinal permeability and absorptive capacity data were determined to be normally distributed by performing a Shapiro‐Wilk test and visually assessing density, histogram, and QQ plots for both data and residuals. The data was assessed for homogeneity using a Levene's test. One‐way ANOVAs were used to determine the effects of age class on intestinal permeability and absorptive capacity while blocking for sex, sample date, and BW. After the ANOVA showed that intestinal permeability was only significantly affected by age, and absorptive capacity only significantly affected by sex, a Welch's t‐test was used without the nonsignificant confounding factors. Welch's t‐test was chosen to analyse significance as it has been shown to limit Type 1 errors and remains rather robust even in cases of violations of normality and homoscedasticity (Delacre et al. 2017). Results are presented as mean ± SD. Statistical significance was defined at *p* < 0.05 and trends at *p* < 0.1.

## 3 Results

### 3.1 Intestinal Permeability and Absorptive Capacity

**Figure 2:**
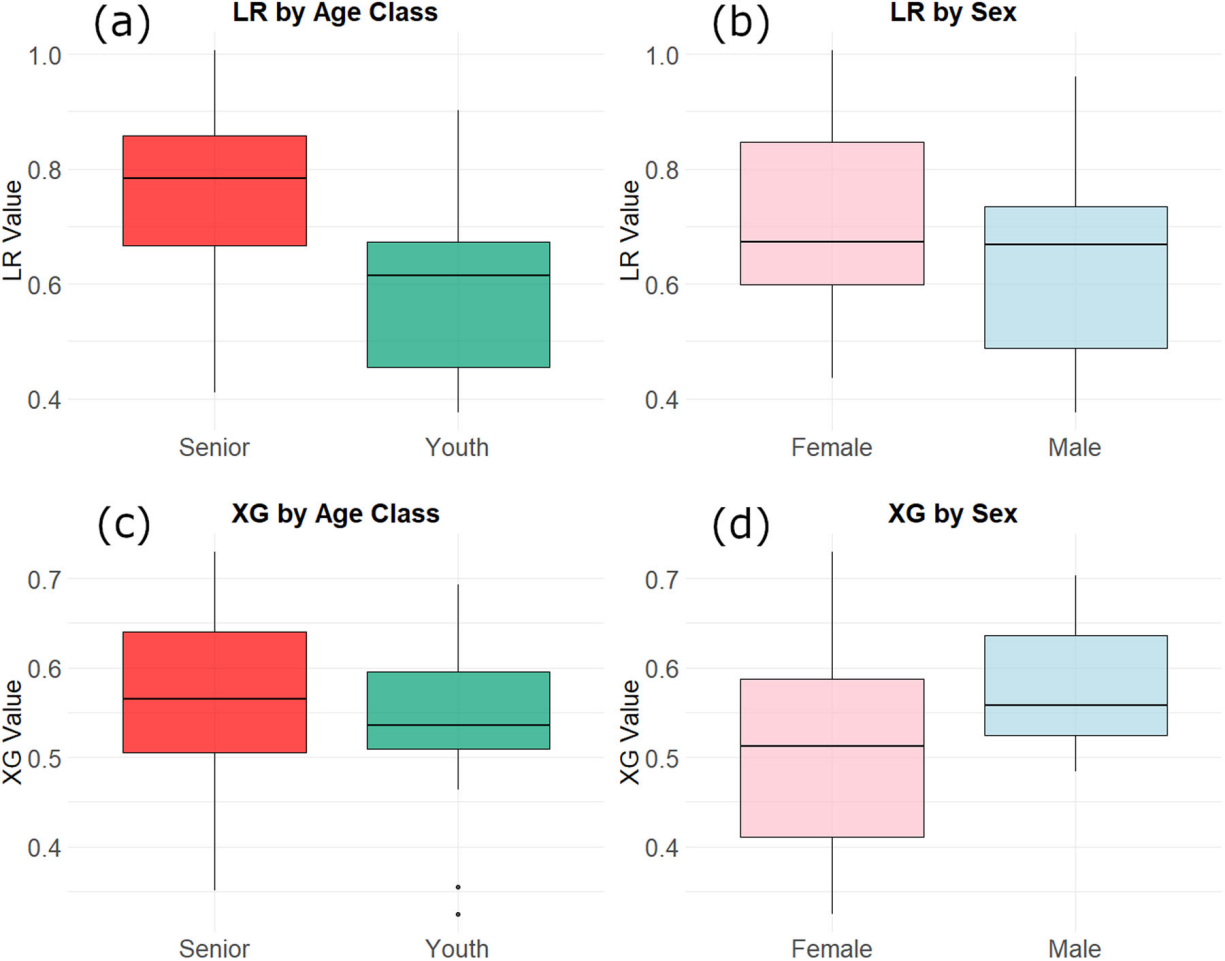
Ratios of lactulose to rhamnose (LR) and xylose to 3‐O‐methylglucose (XG) in plasma of domestic cats. (a) LR for senior (*n* = 15) and young (*n* = 21) cats (*p* = 0.004), (b) LR for female (*n* = 15) and male (*n* = 21) cats, (c) XG for senior (*n* = 15) and young (*n* = 21) cats, (d) XG for female (*n* = 15) and male (*n* = 21) cats (*p* = 0.033). [Color figure can be viewed at wileyonlinelibrary.com]

Intestinal permeability, represented as the LR ratio, was higher (*p* = 0.004) in senior cats (0.75 ± 0.160) than young cats (0.59 ± 0.139) (Figure 2), but not different (*p* = 0.288) between male (0.63 ± 0.161) and female (0.70 ± 0.170) cats. There were no significant effects observed for sampling date (*p* = 0.652) or BW (*p* = 0.951). Absorptive capacity, represented as the XG ratio, was not different (*p* = 0.440) between senior (0.56 ± 0.113) and young cats (0.54 ± 0.089). There were no significant effects observed for sample date (*p* = 0.580) or BW (*p* = 0.652). Sex, however, had a significant (*p* = 0.033) effect on the ratio of XG, being higher in male cats (0.58 ± 0.070) compared to female cats (0.50 ± 0.117). This effect was not significant within the senior group between the males (0.61 ± 0.075) and females (0.51 ± 0.130) (*p* = 0.105) or the young group between the males (0.56 ± 0.063) and females (0.50 ± 0.114) (*p* = 0.173).

### 3.2 Complete Blood Count

Based on CBC analysis, all cats had values for each blood component within the normal range so were considered to be healthy. Segmented neutrophil count (*p* = 0.033) was positively correlated with the senior age class, while haemoglobin (*p* = 0.001), HCT (*p* = 0.010), MCH (*p* = 0.011), platelets (*p* = 0.031), and lymphocyte count (*p* = 0.006) were negatively correlated with the senior age class. MCHC (*p* = 0.010) was positively correlated with LR, and there was a negative trend between MCV and LR (*p* = 0.055). There was a negative trend between RBC and XG (*p* = 0.062) and a positive trend between MCV and XG (*p* = 0.081). See supplementary information for correlation figures (Figures S1–S10).

## 4 Discussion

To the authors' knowledge, this is the first study to use the SAT and discover intestinal permeability is greater, but absorptive capacity is similar in older cats as compared to young cats. Further, while permeability was similar, absorptive capacity was greater in male cats as compared to female cats when analysing the whole study population. This new knowledge of physiological changes associated with ageing in cats provides a new area to further research and potentially improve healthy ageing.

The higher intestinal permeability in older cats observed in the current study supports the hypothesis that the feline GIT undergoes age‐related alterations. This contrasts with a study conducted in dogs, which did not find an increase in intestinal permeability with age (Garden et al. 1997). While there is documented evidence of increased intestinal permeability in Drosophila (Clark et al. 2015), rats (Katz et al. 1987), mice (Thevaranjan et al. 2017), and baboons (Tran and Greenwood‐Van Meerveld 2013) using various methods, studies in humans have reported no such change (Saltzman et al. 1995; Wilms et al. 2020).

The higher intestinal permeability observed in senior cats could be indicative of inflammation in the GIT, leading to infiltration of pathogens and toxins into the periphery. Senior cats over the age of ten have significantly lower RBC, Hb, HCT, WBC, lymphocyte counts, and eosinophil counts than younger adult cats (Campbell et al. 2004; Czarnecki‐Maulden et al. 2004) and generally agrees with the present study. A decrease in these parameters can be indicative of immunosenescence, the gradual decline of the efficacy of the immune system as a natural part of the ageing process (Franceschi et al. 2007). These findings suggest age‐related changes in cats' immune system, which may impact their ability to respond to infections and immune challenges as they get older. The chronic, low‐level inflammation that accompanies ageing in humans and other mammals, referred to as inflammageing, is also thought to manifest in cats, suggesting a potential link between inflammation and the disruption of TJ, leading to increased intestinal permeability during states of inflammation (Karper 2011; McKenzie 2022). While not measured in the current study, there have been age‐related increases in intestinal permeability in humans through the presence of inflammatory and TJ protein biomarkers associated with mucosal barrier and TJ integrity and function (Man et al. 2015). The heightened inflammation typically observed in older animals and indicated in this study through an increased segmented neutrophil count and decreased lymphocyte count might be responsible for the increased intestinal permeability observed in the cats or might be a result of it. Increased neutrophil counts have been seen with age in humans and mice, and are commonly associated with inflammation and observed to stimulate cellular senescence, further exacerbating systemic inflammation (Kristof Van Avondt et al. 2023).

In the current study, age did not affect absorptive capacity in the cat. However, while sex difference was not a primary outcome of the current study, absorptive capacity was found to be higher in male compared to female cats. While there are no specific studies assessing absorptive capacity by sex in the cat, a study involving dogs with chronic enteropathies found no difference in absorptive capacity or intestinal permeability between sexes using urinary LR and XG ratios, which have been found comparable to plasma and serum ratios in multiple studies (Fleming et al. 1996; Allenspach et al. 2006; Bruet et al. 2008). Sex may also play a role in influencing the integrity and functionality of the small intestinal barrier. Previous studies in rats found reduced intestinal permeability during oestrus, and greater permeability in the following luteal phase, but this was not assessed in the current study (Homma et al. 2005; Braniste et al. 2009). Females also tend to possess a more diverse gut microbiome that is linked with reduced intestinal permeability compared to males in humans (Edogawa et al. 2018). In a 2014 study involving 4009 cats, female cats (15.0 years; IQR 11.0–17.4) had a higher median longevity than male cats (13.0 years; IQR 7.6–16.0) which was statistically significant (*p* < 0.001) (O'Neill et al. 2015). The authors of this study did not delve further into possible explanations behind this, allowing for speculation that microbial diversity could be a contributing factor.

The dose used in the present study follows the validated method as used in Patterson et al. (2024). The solution was hyperosmolar to better discriminate between healthy and impaired intestinal barriers, however a hyperosmolar solution can also cause increased permeation of lactulose (Travis and Menzies 1992; Uil et al. 2000). While the osmolarity of the solution administered did not reach the level of hyperosmolarity shown to enhance lactulose permeation (Travis and Menzies 1992), the choice of a hyperosmolar solution, unlike the iso‐osmolar approach used in prior cat studies (Papasouliotis et al. 1993; Bijlsma et al. 1995), might clarify the higher LR ratio observed in the present study. This discrepancy, however, makes it challenging to directly compare the results to previous research on feline intestinal permeability. Nevertheless, it paves the way for further exploration in this field as the hyperosmolar dose is able to be given to most cats without sedation and by using low‐stress handling techniques.

As one of the few intestinal permeability or absorptive capacity trials conducted in cats, and the only one to compare these measurements across age class and sex, these results offer a unique and comprehensive insight into feline gastrointestinal physiology, clearly suggesting an increase in intestinal permeability due to ageing and differences in intestinal absorption between males and females. Since the present study was limited on the number of entire male and female cats, further research should focus on determining the relationship that intestinal permeability has with neuter status, as well as other aspects of ageing, such as reduced nutrient digestibility, immunosenescence, and loss of microbial diversity.

## 5 Conclusions

In conclusion, senior cats had significantly greater intestinal permeability values when compared to younger cats, suggesting age‐related changes in their GIT. The correlations of intestinal permeability with markers of inflammation suggest that inflammation may play a role in these changes or be affected by them. Further exploration of biomarkers of intestinal health and pro‐inflammatory cytokines may provide additional insight into the relationship between intestinal permeability and inflammation. This study significantly contributes to the pool of knowledge of feline gastrointestinal physiology, shedding light on age and sex‐related differences in haematological parameters, intestinal permeability, and absorptive capacity.

## Conflicts of Interest

The authors declare no conflicts of interest.

## Supporting information


**Figure S1:** Segmented neutrophil count is positively correlated with the senior age class (p = 0.033) and positively correlated with continuous age (p = 0.014). **Figure S2:** Haemoglobin is negatively correlated with the senior age class (p = 0.001) and negatively correlated with continuous age (p < 0.001). **Figure S3:** Haematocrit (HCT) is negatively correlated with the senior age class (p = 0.010) and negatively correlated with continuous age (p = 0.005). **Figure S4:** Mean corpuscular haemoglobin (MCH) is negatively correlated with the senior age class (p = 0.011) and negatively correlated with continuous age (p = 0.004). **Figure S5:** Platelet count is negatively correlated with the senior age class (p = 0.031) and negatively correlated with continuous age (p = 0.015). **Figure S6.** Lymphocyte count is negatively correlated with the senior age class (p = 0.006) and negatively correlated with continuous age (p = 0.003). **Figure S7:** Mean corpuscular haemoglobin (MCHC) is positively correlated with intestinal permeability (LR) (p = 0.010). **Figure S8:** Mean corpuscular volume (MCV) is negatively correlated with intestinal permeability (LR) (p = 0.055). **Figure S9:** Red blood cell count (RBC) is negatively correlated with absorptive capacity (XG) (p = 0.062). **Figure S10:** Mean corpuscular volume (MCV) is positively correlated with absorptive capacity (XG) (p = 0.081).

## Data Availability

The data that support the findings of this study are available from the corresponding author upon reasonable request. The data that support the findings of this study are available from the corresponding author, KP, upon reasonable request.
