# Supplementary material for: Age‐Dependent Increase in Small Intestinal Permeability and Sex‐Dependent Absorptive Capacity in Cats (Felis catus)
Source: J Anim Physiol Anim Nutr (Berl). 2025 Nov 2;110(2):169–76. doi: 10.1111/jpn.70015 (PMC13001013; doi:10.1111/jpn.70015)
Supplement: Supplementary file 1 — Figure S1: Segmented neutrophil count is positively correlated with the senior age class (p = 0.033) and positively correlated with continuous age (p = 0.014). Figure S2: Haemoglobin is negatively correlated with the senior age class (p = 0.001) and negatively correlated with continuous age (p < 0.001). Figure S3: Haematocrit (HCT) is negatively correlated with the senior age class (p = 0.010) and negatively correlated with continuous age (p = 0.005). Figure S4: Mean corpuscular haemoglobin (MCH) is negatively correlated with the senior age class (p = 0.011) and negatively correlated with continuous age (p = 0.004). Figure S5: Platelet count is negatively correlated with the senior age class (p = 0.031) and negatively correlated with continuous age (p = 0.015). Figure S6. Lymphocyte count is negatively correlated with the senior age class (p = 0.006) and negatively correlated with continuous age (p = 0.003). Figure S7: Mean corpuscular haemoglobin (MCHC) is positively correlated with intestinal permeability (LR) (p = 0.010). Figure S8: Mean corpuscular volume (MCV) is negatively correlated with intestinal permeability (LR) (p = 0.055). Figure S9: Red blood cell count (RBC) is negatively correlated with absorptive capacity (XG) (p = 0.062). Figure S10: Mean corpuscular volume (MCV) is positively correlated with absorptive capacity (XG) (p = 0.081). [file JPN-110-169-s001.docx]

Figure S1. Segmented neutrophil count is positively correlated with the senior age class (p = 0.033) and positively correlated with continuous age (p = 0.014).


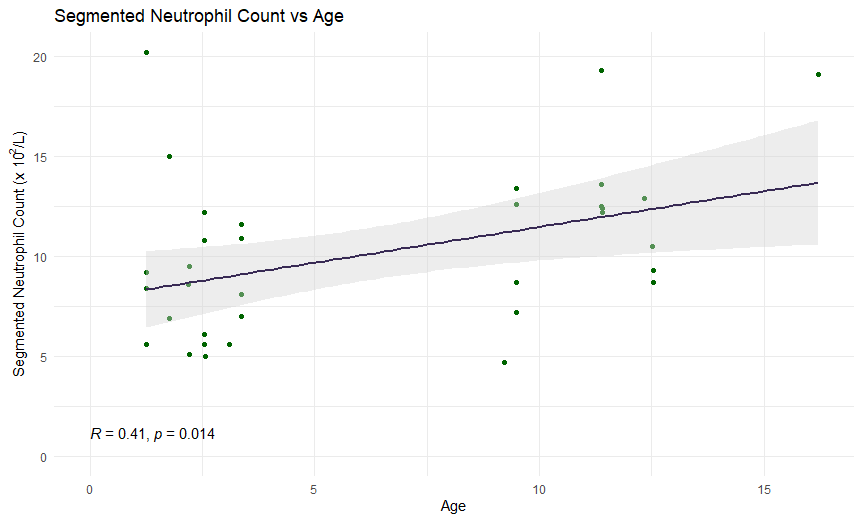


Figure S2. Haemoglobin is negatively correlated with the senior age class (p = 0.001) and negatively correlated with continuous age (p < 0.001).


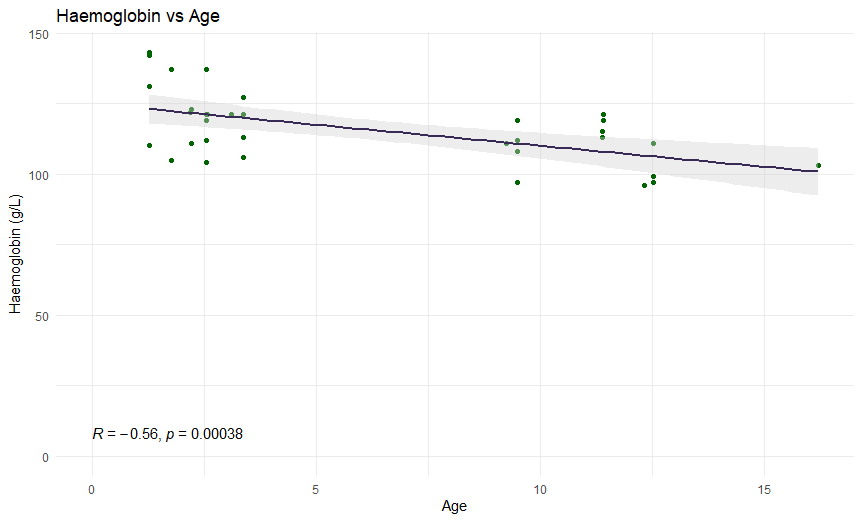

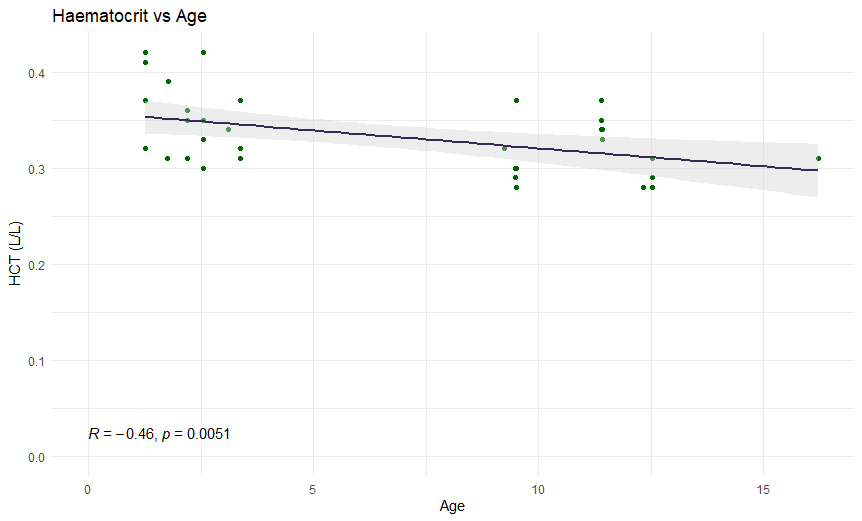


Figure S3. Haematocrit (HCT) is negatively correlated with the senior age class (p = 0.010) and negatively correlated with continuous age (p = 0.005).


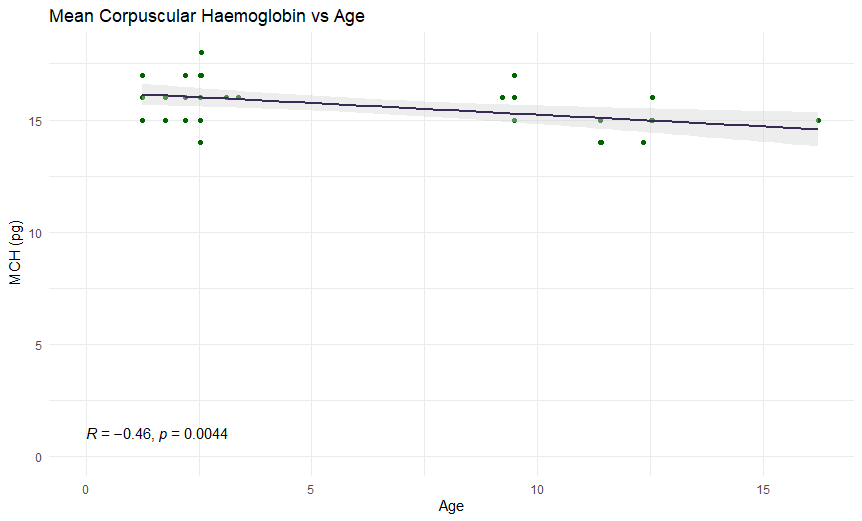


Figure S4. Mean corpuscular haemoglobin (MCH) is negatively correlated with the senior age class (p = 0.011) and negatively correlated with continuous age (p = 0.004).


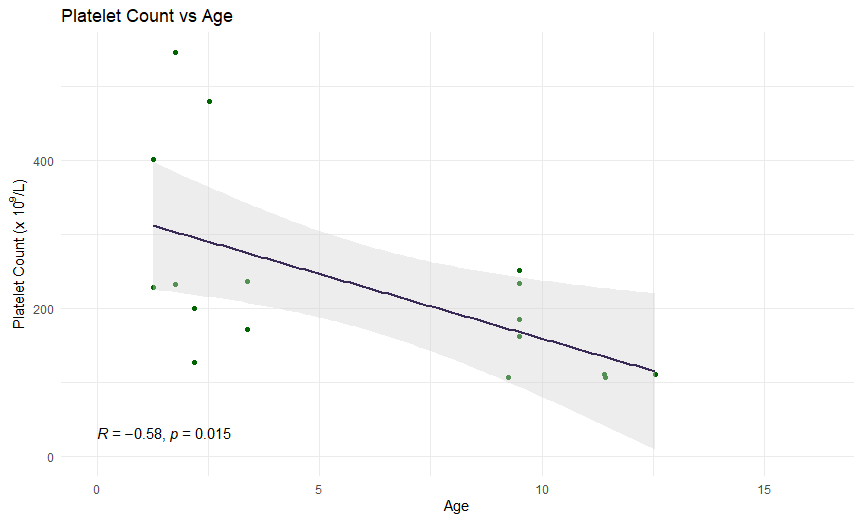


Figure S5. Platelet count is negatively correlated with the senior age class (p = 0.031) and negatively correlated with continuous age (p = 0.015).

Figure S6. Lymphocyte count is negatively correlated with the senior age class (p = 0.006) and negatively correlated with continuous age (p = 0.003).


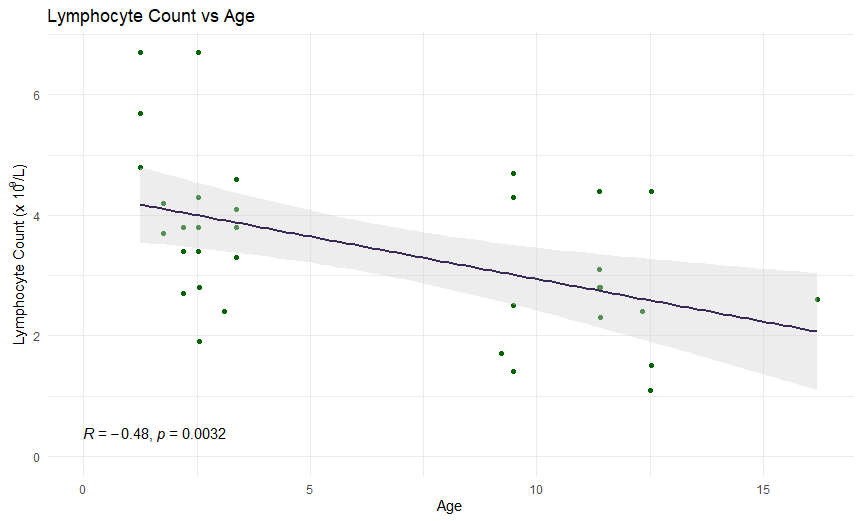

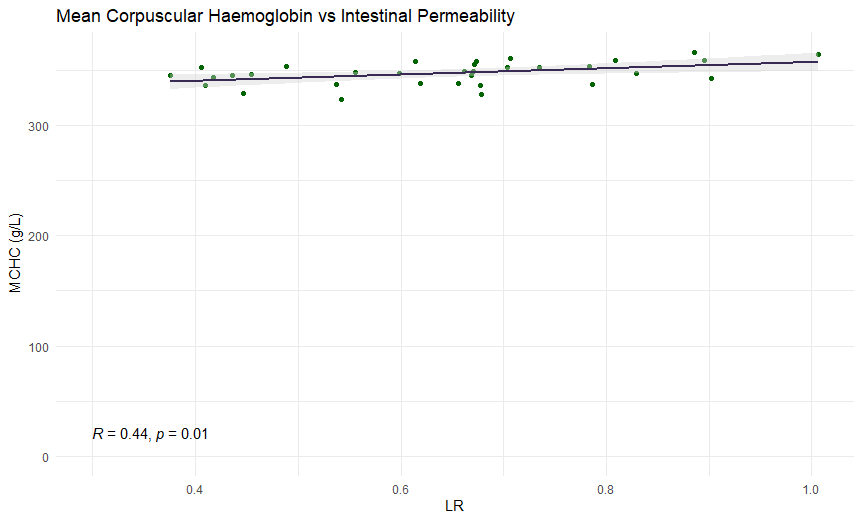


Figure S7. Mean corpuscular haemoglobin (MCHC) is positively correlated with intestinal permeability (LR) (p = 0.010)

Figure S8. Mean corpuscular volume (MCV) is negatively correlated with intestinal permeability (LR) (p = 0.055)


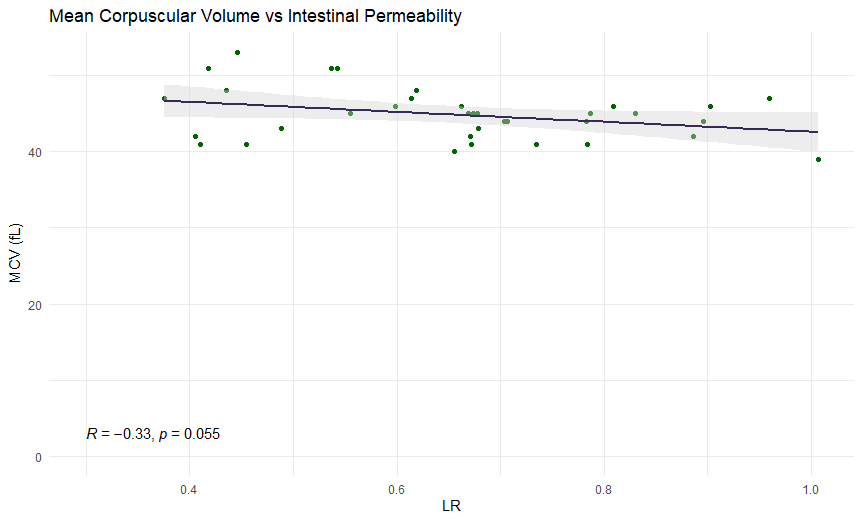

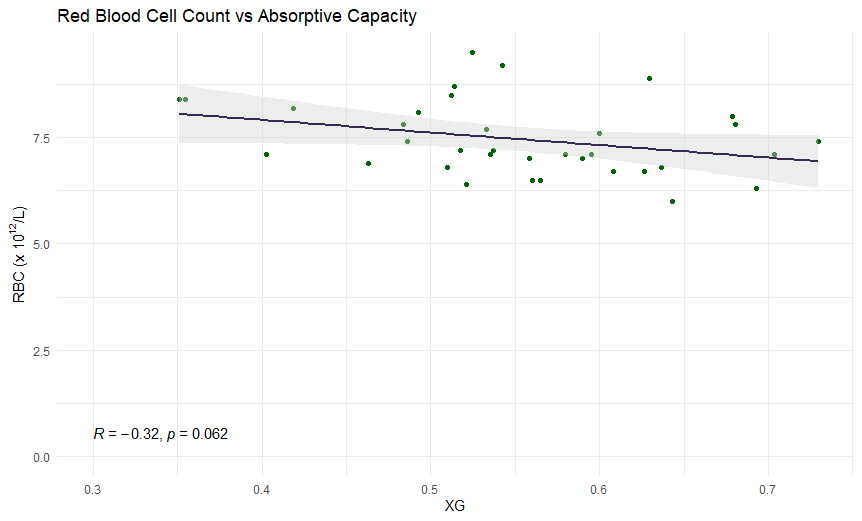


Figure S9. Red blood cell count (RBC) is negatively correlated with absorptive capacity (XG) (p = 0.062)


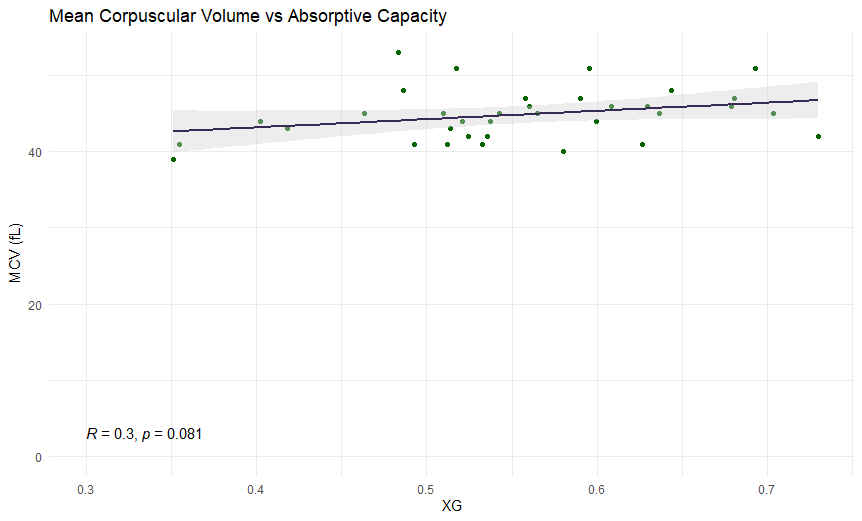


Figure S10. Mean corpuscular volume (MCV) is positively correlated with absorptive capacity (XG) (p = 0.081)
